# Enzyme-derived deer velvet extract activate the immune response in cyclophosphamide-induced immunosuppressive mice

**DOI:** 10.1007/s10068-023-01275-4

**Published:** 2023-02-21

**Authors:** Sinhwa Baek, Cho I Park, Yun Gyeong Hwang, Hyejin Jeon, Seong-Eun Kim, Aeri Song, Hyun-Je Park, Ilbum Park, Jongsoo Kang, Joo Young Cha

**Affiliations:** 1 Yuhan Care Co., Ltd, Yuhan Care R&D Center, Yongin, 17084 Republic of Korea; 2 Yuhan Care Co., Ltd, Yuhan Natural Product R&D Center, Andong, 36618 Republic of Korea; 3 Yuhan Care Co., Ltd, Seoul, 07335 Republic of Korea

**Keywords:** Deer velvet, Immune-enhancing effect, Functional food, Immunosuppressed mouse model, Natural killer cell activity

## Abstract

Deer velvet (DV) is an oriental traditional medicine used to treat various diseases. The present study examined the effect of flavourzyme-derived DV extract (YC-1101) on macrophages and an immunosuppressed mouse model. YC-1101 induced activation of macrophages as measured by nitric oxide production, cell proliferation, and cytokine release via concentration-dependent phosphorylation of c-Jun N-terminal kinase, extracellular signal-regulated kinase, and AKT, and nuclear translocation of p65 in macrophages. In addition, oral YC-1101 administration significantly increased splenocyte proliferation and natural killer cell activity in the immunosuppressed mouse model. Moreover, the levels of immune-related cytokines such as tumor necrotic factor-α, interferon-γ, and interleukin-2 were significantly increased by YC-1101 treatment comparable to the control group. Thus, these results suggest that YC-1101 is an efficient natural ingredient that has an immune-enhancing effect, and it might be a potential functional food for improving immunity.

## Introduction

The human immune system consists of innate and adaptive immune responses that cooperate efficiently to maintain immune homeostasis. However, owing to rapid industrialization and environmental changes, the prevalence of immune-related diseases has continuously increased recently (Wang et al., 2022). Although various drugs and functional foods are being developed to prevent and improve immune-related diseases, most have limited efficacies and severe side effects (Saurin et al., 2022).

It is well known that innate immunity plays a pivotal role in destroying pathogens by regulating several immune cells, such as neutrophils, mononuclear cells, and natural killer (NK) cells (Ham et al., 2022; Ye et al., 2022). In addition, macrophages secrete a variety of cytokines during the identification and removal of bacteria or foreign substances (Bueno-Silva et al., 2022). NK cells directly kill cancer cells or infected cells and secrete cytokines to activate dendritic cells (Rahimian et al., 2022).

The spleen is a major lymphatic organ that collects antigens from the blood and is involved in the maturation of B cells and stimulation of T cells (Lewis et al., 2019). The proliferation of lymphocytes in the spleen and secretion of cytokines, such as interferon (IFN)-γ, tumor necrosis factor (TNF)-α, interleukin (IL)-1β, and IL-6, are involved in immune regulation (Hernandez et al., 2021; Ioanna et al., 2021). These cytokines are used as signaling molecules to control and stimulate the body’s defense system. They also play pivotal roles in immune regulation, treatment of infectious diseases, hematopoietic development, tissue recovery, and cell growth (Steen et al., 1998; Teijaro, 2017). IFN-γ has a priming effect on macrophages to secrete for higher levels of proinflammatory cytokines and enhance the microbicidal and tumoricidal activity of macrophages (Cong et al., 2014).

Deer velvet (DV) generally refers to dried antlers from dehaired young deer that have not yet ossified or are only slightly ossified (Yoo et al., 2022). DV is largely provided by *Cervus nippon* Temminck, *Cervus elaphus* Linnaeus, or *Cervus canadensis* Erxleben (Kim et al., 2020) and is divided into the top, upper, middle, and base parts, which contain 16 amino acids (Zhang et al., 2019). DV contains high levels of proline, glycine, and glutamic acid (Liu et al., 2020). Amino acids, collagen, chondroitin, glucosamine, ganglioside, and hyaluronic acid have been identified as physiologically active components of DV (Dai et al., 2011). DV exerts several pharmacological effects, such as tonicity, growth and development promotion, blood retention, hematopoiesis, organ enhancement, physical function enhancement, and physical vitality enhancement (Wu et al., 2013). Recent studies have identified multiple effects of DV on the liver, immune system, cardiovascular system, aging, stress, sugar metabolism, and hematopoiesis (Chen et al., 2014; He et al., 2022).

In this study, we demonstrated the immune-enhancing effects of YC-1101, an enzymatically digested DV extract, by analyzing cytokine levels and NK cell activity in cyclophosphamide (CP)-induced immuno-suppressed mice. We also investigated the biological mechanisms of action of YC-1101 in RAW 264.7 cells.

## Materials and methods

### Preparation of YC-1101

DV freeze-dried (FD) powder was manufactured from DV, classified as *Cervus elaphus* Linnaeus in New Zealand. DVFD powder was commercially purchased from Alpine Deer New Zealand Limited Partnerships (Tauranga, New Zealand). For the manufacture of YC-1101, DVFD powder was mixed with water and the mixtures were heated to 50 °C. Flavourzyme (Daejongzymes, Seoul, Republic of Korea) solution was then added, and the digestion mixtures were heated and mixed over 12 h. Enzyme deactivation was performed by heating the mixtures over 90 °C at least 20 min. After cooling, the enzyme digests were centrifuged over 200 × *g* at 25 °C. Each supernatant was filtered under vacuum through Celite 545. Finally, the extracts were prepared by freeze-drying after concentration and sterilization until leaving behind a moisture contents under 8%. YC-1101 (production code name: YHC-BE-2040) was analyzed by the HPLC method to confirm that it contains 0.7–1.1 mg/g of uracil (data not shown). HPLC method was performed using an Waters Alliance LC (Waters Corp., Milford, MA, USA) equipped with a quaternary gradient pump and a sample manager. HPLC method was developed using NH2 column (Waters spherisorb NH2, 4.6 mm × 250 mm, 4.6 μm) which was maintained at 30 ℃. Twenty microliters of sample or standard solution was injected into the column. The gradient elution system consisted of 90% of acetonitrile with 50 mM ammonium acetate (pH 5.6) (solvent A) and 50% acetonitrile with 10 mM ammonium acetate (pH 5.6) (solvent B). The separation was achieved using the following gradient: 0–10 min, 0% B; 10–12 min, 0–34% B; 12–17 min, 34–64% B; 17–25 min, 64–100% B; 25–35 min, 100% B; 35–37 min, 100–0% B, 37–47 min, 0% B. The flow rate was 0.7 mL/min, and the eluate was monitored at 254 nm by photodiode array detector. The content of uracil in YC-1101 was calculated using standard curve of uracil (Sigma-Aldrich, St. Louis, MO, USA).

To compare the activity of YC-1101, YHC-BE-2038 has been prepared without enzyme digestion process. DVFD powder (10 g) was mixed with water (200 mL) in a round-bottom flask. A condenser was fitted, and the mixture was boiled under reflux for 3 h at 100 °C. After cooling, the mixture was centrifuged over 200×*g* at 25 °C. Water was added to the supernatant to a volume of 200 mL, and the mixture was then filtered under vacuum through Celite 545. Finally, the extract was prepared by freeze-drying after concentration and sterilization until leaving behind a moisture contents under 8%.

YHC-T-2105 has been used as a positive control, which contains ginsenoside Rg1, Rb1, and Rg3. This extract is made of freeze-dried powder of the red ginseng concentrate purchased at Gimpo Paju Ginseng Agricultural Cooperative (Paju, Republic of Korea).

### In vitro* cell culture*

A mouse macrophage cell line (RAW 264.7) was maintained in Dulbecco’s Modified Eagle’s Medium (DMEM; Gibco BRL, Grand Island, NY, USA) containing fetal bovine serum and antibiotic–antimycotic (penicillin, streptomycin, and amphotericin B). The cells were stimulated with and without YC-1101 or YHC-BE-2038 (4–500 µg/mL). Lipopolysaccharide (LPS, 1 μg/mL, Sigma-Aldrich) was used as a positive control.

### Cell proliferation assay

Cell proliferation was assessed using a CCK-8 kit (Dojindo, Kumamoto, Japan). Cells (5 × 10^3^ cells/well) were seeded in a 96-well plate and then treated with samples after 24 h. Before 10 μL of CCK-8 (Dojindo) were added to each well, cells were incubated for 2 days. Cells were then incubated for 1 h at 37 °C in a 5% CO_2_ incubator. The absorbance of each well was measured at 450 nm using a microplate reader (Infinite 200 Pro; TECAN, Männedorf, Switzerland). The results are given as a percentage relative to the untreated control.

### Assessment of nitric oxide (NO) production

After incubating RAW 264.7 cells for 24 h, the medium was replaced with medium containing deer velvet extract (DVE). To estimate NO and cytokine production, the supernatants of cultured RAW 264.7 cells were separated and centrifuged at 800×*g* for 5 min. NO levels were determined using an NO detection kit (Enzo Life sciences, Farmingdale, NY, USA), a common experimental technique for assessing nitrite. The absorbance was measured at 540 nm using a microplate reader, and NO production was quantified using four-parameter logistic regression of the standard curve.

### Real-time polymerase chain reaction

After culturing RAW 264.7 cells (5 × 10^5^ cells/well) in six-well plates for 24 h, the medium was replaced with medium containing the negative control or each test substance at different concentrations, followed by further incubation. Total cellular RNA was extracted from cells using an RNA extraction kit (Qiagen, Hilden, Germany). PCR was performed in a final volume of 16 µl containing 20 ng/µl cDNA template, 3 µmol each of forward and reverse primers, and RNase-free water. PCR was performed using a TOPreal One-step kit (Enzynomics, Daejeon, Republic of Korea). The TNF-α, IL-6, IL-1β, inducible NO synthase (iNOS), cyclooxygenase-2 (COX-2), and β-actin genes were reverse-transcribed at 50 °C for 30 min, pre-denaturated at 95 °C for 15 min, and then subjected to 45 cycles of denaturation, annealing, and elongation (95 °C for 5 s and 60 °C for 30 s). The primers used are listed in Table 1. β-actin was used as the housekeeping gene, and gene expression was normalized using the 2^−ΔΔ^CT method.

**Table 1:** Primer sequences for real-time polymerase chain reaction

Gene	Primer	Sequences
TNF-α	Forward	CTGTAGCCCACGTCGTAGC
	Reverse	TTGAGATCCATGCCGTTG
IL-6	Forward	CTTCCATCCAGTTGCCTTCT
	Reverse	CTCCGACTTGTGAAGTGGTATAG
IL-1β	Forward	CAAGGAGAACCAAGCAACGA
	Reverse	GGGTGTGCCGTCTTTCATTA
iNOS	Forward	ACATCGACCCGTCCACAGTAT
	Reverse	CAGAGGGGTAGGCTTGTCTC
COX-2	Forward	CCAGCACTTCACCCATCAGTT
	Reverse	ACCCAGGTCCTCGCTTATGA
β-actin	Forward	CAGCCTTCCTTCTTGGGTATG
	Reverse	GGCATAGAGGTCTTTACGGATG

### Western blotting

RAW 264.7 cells were treated with YC-1101 or YHC-BE-2038 for 30 min. The cells were lysed in 300 µl of RIPA buffer containing a protease/phosphatase inhibitor cocktail. Lysed protein samples were separated by 4–20% sodium dodecyl sulfate–polyacrylamide gel electrophoresis and transferred to polyvinylidene fluoride membranes. The membranes were blocked in 5% bovine serum albumin (BSA) for 1 h and incubated with primary antibodies overnight at 4 °C. The membranes were next treated with horseradish peroxidase (HRP)-conjugated secondary antibodies for 1 h at room temperature (22 ± 2 °C). Finally, the membranes were washed in tris-buffered saline with 0.1% Tween 20 detergent (TBST) and then reacted with enhanced chemiluminescence detection reagents. The protein bands on membranes were visualized using the ChemiDoc Imaging System (Bio-Rad, Hercules, CA, USA).

### Immunofluorescence staining

RAW 264.7 cells were plated on Lab-Tek chamber slides (Nunc, Sigma-Aldrich). Cells were treated with YC-1101 for 120 min, washed with phosphate buffered saline (PBS; Welgene, Gyeongsan, Republic of Korea), and fixed in 4% paraformaldehyde for 20 min at room temperature (22 ± 2 °C). Cells were next washed with PBS, permeabilized in cold methanol for 30 min at − 20 °C and washed several times with PBS. Cells were blocked in 1% BSA for 1 h and incubated with antibodies overnight at 4 °C. Finally, cells were washed in PBS and covered with Prolong antifade solution containing 4',6-diamidino-2-phenylindole (DAPI). Cells were imaged using an EVOS M7000 imaging system (Thermo Fisher Scientific, Waltham, MA, USA).

### Experimental animals

Specific pathogen-free 6-week-old C57BL6 male mice (Koatech, Pyeongtaek, Republic of Korea) were housed in a specific pathogen-free environment in an animal facility for at least 1 week before the experiments. Six mice per group were used in the experiment. The breeding room was maintained under the following conditions: temperature, 20–26 °C; relative humidity, 40–70%; ventilation, 12 times/h; illumination, from 8:00 a.m. to 8:00 p.m.; illuminance, 150–300 Lux. Water and food (+ 40 RMN, SAFE complete care competence, Augy, France) were provided ad libitum. The research protocol was approved by the Institutional Animal Care and Use Committee and conducted according to principles clearly stated in the “Animal Care Act” prepared by the Ministry of Agriculture and Forestry, Republic of Korea. This study was approved by the Animal Ethics Committee Chonbuk National University Hospital Non-Clinical Evaluation Center, CBNUHNCEC (JBNUH-IACUC-2022-04, January 18th, 2022).

### CP-induced mice model

CP (Sigma-Aldrich) was dissolved in water for injection, and mice were intraperitoneally injected with CP 70 mg/kg body weight (BW)/day 1–3 d before DVE treatment and on day 13 of DVE treatment. Each day on days 1–14, YC-1101 was orally administered at dosage of 50, 62.5, and 100 mg/kg BW/day. As a positive control, YHC-T-2105 was orally administered at dosages of 3 mg/g BW/day as a combination of ginsenoside Rg1, Rb1, and Rg3 in Korean red ginseng extract. Mice were sacrificed after 2 weeks, and their blood and spleens were collected for cytokines measurement, splenocyte proliferation assays, or NK cell activity assays.

### Splenocyte isolation

The separated spleen was washed twice with RPMI 1640 medium (Gibco, Thermo Fisher Scientific) contained 100 U/mL of penicillin (Gibco, Thermo Fisher Scientific) and 100 mg/mL of streptomycin (Gibco, Thermo Fisher Scientific). To separate into single cells, add the red blood cell lysis buffer (Sigma-Aldrich) for 10 min. After washing with RPMI 1640 medium, the splenocyte suspension was cultured using RPMI 1640 medium.

### Splenocyte proliferation assay

Primary splenocytes were isolated from C57BL6 mouse spleens. Cells were seeded in 96-well plates at 3 × 10^6^ cells/well. After stimulation with LPS or concanavalin A (Con A, Sigma-Aldrich) for 48 h, the 3-(4,5-dimethylthiazol-2-yl)-5-(3-carboxymethoxyphenyl)-2-(4-sulfophenyl)-2H-tetrazolium (MTS; Promega, Madison, USA) assay was performed. The product was determined using a microplate reader at 490 nm, and cell proliferation was calculated in reference to the CP group.

### NK cell activity assay

Isolated splenocytes were used as effector cells, and YAC-1 cells (mouse lymphoma cell line) were used as the target cells. Effector and target cells were mixed and incubated at an effector:target ratio of 100 (1 × 10^6^ cells/well):1 (1 × 10^4^ cells/well). After incubation for 4 h, NK cell activity cell was measured using lactate dehydrogenase (LDH) cytotoxic assay kit (Abcam, Cambridge, UK).

### Cytokine measurement

Whole blood was collected from mice and then centrifuged at 800×*g* for 10 min and stored at − 80 °C. The levels of TNF-α, IFN-γ, IL-6, and IL-2 were determined using ELISA kits (Cusabio, Wu Han, China) according to the manufacturer’s instructions.

### Statistical analysis

All in vitro experiments were repeated at least three times with consistent results. The levels of significance for comparison between samples were determined by an independent samples *t*-test or Dunnett’s *t*-test using statistical software for in vitro data. In vivo data were compared between the control and CP groups using the nonparametric Kruskal–Wallis test followed by Dunn's post hoc multiple comparison test (SPSS Version 21.0 Inc., Chicago, IL, USA). The data in the graphs are expressed as the mean ± standard error of the mean (SEM), and *p* < 0.05 denoted statistical significance. The statistical package of Graph pad prism version 8.0 (GraphPad Software Inc., La Jolla, CA, USA) was used in the analysis.

## Results and discussion

### YC-1101 induced cell proliferation and NO production in RAW 264.7 macrophages

**Fig. 1:**
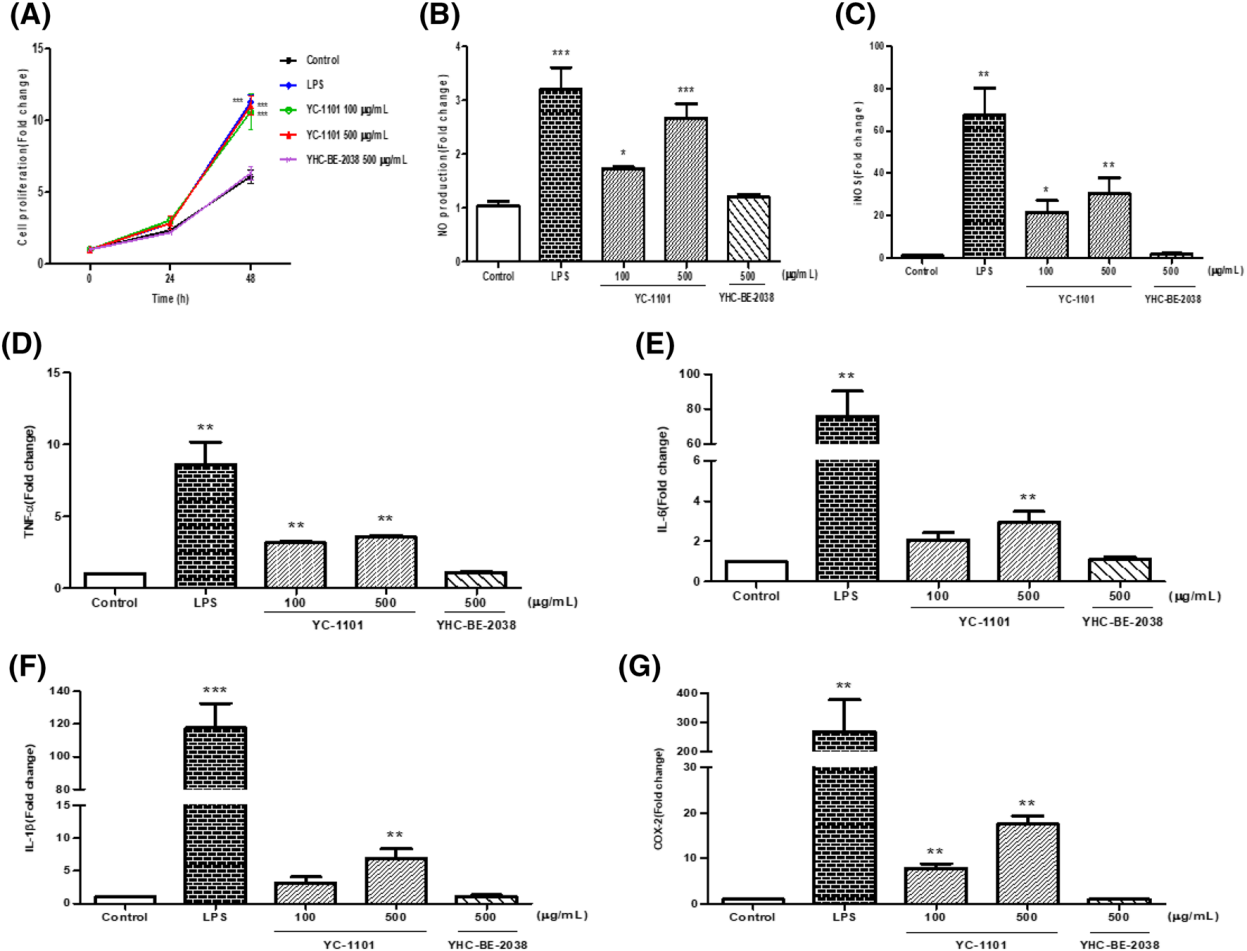
Effects of YC-1101 on cell proliferation (**A**), NO production (**B**), iNOS expression (**C**), and macrophage-related cytokine production (**D**–**G**) in RAW 264.7 cells. Cells were treated with YC-1101 (100 or 500 μg/mL) or YHC-BE-2038 (500 μg/mL). LPS (1 μg/mL) was used as a positive control. (**A**) RAW 264.7 cell proliferation was measured using commercial kits. (**B**) Supernatants were harvested after treatment with DVE and assayed for NO production. (**C**–**G**) Total cellular RNA was extracted for RT-PCR using iNOS, COX-2, IL-1β, IL-6, and TNF-α primers. β-actin was measured as a loading control. Data are expressed as the mean ± SEM (n = 6). *p < 0.05 vs. control; **p < 0.01 vs. control; ***p < 0.001 vs. control

Macrophages are important phagocytes that act as a bridge between innate and adaptive immunity, with a high capacity for phagocytosis, proinflammatory cytokine production, and antigen presentation (Murray and Wynn, 2012). The proliferation of macrophages was significantly increased by YC-1101 treatment compared with that in the control group (Fig. 1A). In general, cell proliferation is an important biological property of macrophages that, to a certain extent, reflects their activation state (Li et al., 2018). Thus, our in vitro results of the proliferation in RAW 264.7 macrophages by YC-1101 suggest that YC-1101 can activate macrophages in vivo. RAW 264.7 cells treated with LPS or YC-1101 exhibited significantly increased NO production and iNOS mRNA expression (Fig. 1B, C), demonstrating that they play a role in improving the phagocytic capacity of macrophages. NO production in macrophages is used as a representative biomarker for estimating immune enhancement; NO is an important signal transmitter that acts as a defense against tumor cells or infected cells in the immune system (Garthwaite, 2018). NO is produced by iNOS in macrophages, and iNOS is expressed by cytokines, which induce the expression of the iNOS gene (Hwang et al., 2022). LPS, which induce cytokine production in macrophages, were used as a positive control. YC-1101 had similar effects on cell proliferation, NO production, and iNOS mRNA expression to those of LPS. YHC-BE-2038, which was used as a negative control, had no effects on cell proliferation and NO production.

### YC-1101 increased immunomodulatory cytokine levels in RAW 264.7 macrophages

The effects of YC-1101 on various inflammatory mediators of the immune system were compared with those of LPS by RT-PCR. To determine the immune-enhancing effects of YC-1101, TNF-α, IL-6, and IL-1β were measured as immunomodulatory cytokines. COX-2 is an immune mediator that participates in inflammatory responses (Ji et al., 2021). The mRNA levels of TNF-α, IL-6, IL-1β, and COX-2 were significantly enhanced by treatment with YC-1101 and LPS in macrophages (Fig. 1D–G). In particular, YC-1101 treatment increased cytokine levels relative to the control in RAW 264.7 macrophages.

These results suggested that YC-1101 is a potent inducer of cytokine secretion in relation to immune activity. YHC-BE-2038 was used as a negative control and had no effect on immunomodulatory cytokine release.

### Molecular mechanism underlying the immunostimulatory effects of YC-1101

There are many ways to regulate the immune system. The c-Jun N-terminal kinase (JNK) and extracellular signal-regulated kinase (ERK) pathways play important roles in macrophage activation and proliferation (Mosser and Edwards, 2008). AKT, also known as protein kinase B, plays an important role in immune modulation. AKT promotes cytokine secretion and programming in innate immune cells, including macrophages (Zhang et al., 2013). AKT can also control macrophage survival and apoptosis (Boronkai et al., 2009). p65 is a nuclear factor kappa-light-chain-enhancer of activated B cells (NF-κB) that regulates immune response (Cui et al., 2022). JNK and ERK are mitogen-activated protein kinases (MAPKs) that signal to p38. MAPKs recognize LPS stimuli and activate signaling pathways inside the cell, promoting the expression of immune-related cytokines, such as TNF-α and IL-6 (Kang et al., 2021). NF-κB signaling is also activated by stimulation, which is translocated to the nucleus and induces the transcription of genes such as COX-2 and iNOS (Jeon et al., 2013).

**Fig. 2:**
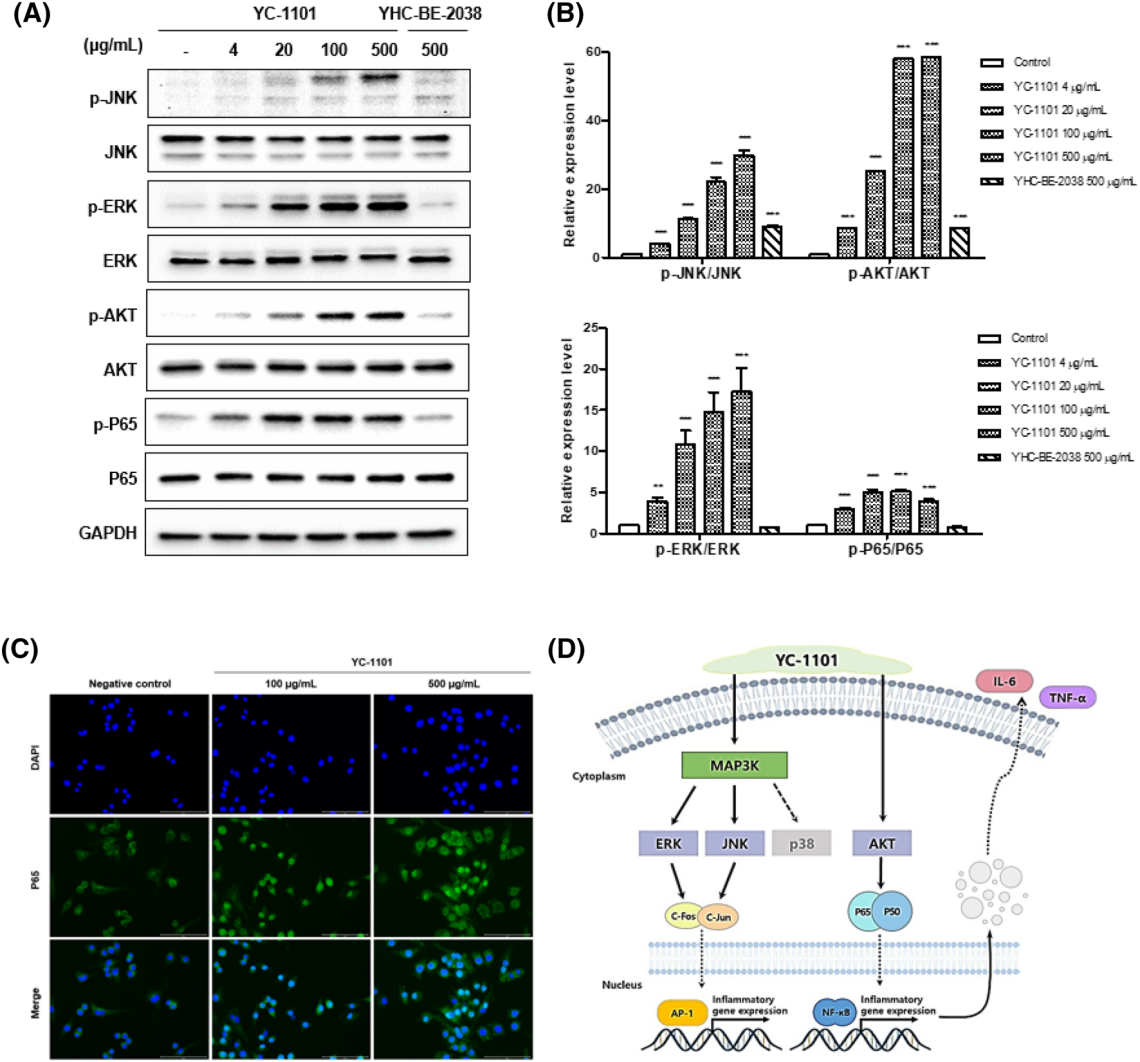
Effects of YC-1101 on biological signaling molecules in macrophage. JNK, ERK, AKT, and p65 phosphorylation were analyzed by western blotting in RAW 264.7 cells (**A**). Western blot quantification analysis (n = 3 per group) (**B**). Effects of YC-1101 on NF-κB p65 translocation in RAW 264.7 cell (**C**). Cells were treated with YC-1101 (100 or 500 μg/mL) and stained with antibodies against NF-κB p65 (shown in green) and DAPI (shown in blue). Subcellular localization of NF-κB p65 was examined using immunofluorescence staining and analyzed using an imaging system. Schematic diagram of the mechanism of action of YC-1101 (**D**). GAPDH was used as the loading control. **p < 0.01 vs. control; ***p < 0.001 vs. control

To investigate the macrophage stimulation related mechanism of YC-1101, we conducted western blot analysis for JNK, ERK, AKT, and p65 signaling in RAW 264.7 cells. As presented in Fig. 2A and B, JNK, ERK, AKT, and p65 were activated by YC-1101 treatment, as indicated by their phosphorylation. These results indicate that YC-1101 activates the JNK, ERK, and AKT pathways and regulates cell proliferation and inflammatory cytokine production (Fig. 2D). To compare the level of NF-κB p65 translocation between the negative control and YC-1101-treated RAW 264.7 cells, immunofluorescence staining was performed and analyzed using an imaging system. As shown in Fig. 2C, p65 was present in the cytoplasm of the negative control group. However, after 120 min of YC-1101 treatment (100 or 500 μg/mL), p65 was increasingly co-localized with DAPI, indicating p65 nuclear translocation.

Therefore, these results demonstrate that the biological mechanism of YC-1101 is associated with the MAPKs, JNK, and NF-kB pathways, which are also triggered by immunostimulants during immune activation by the increased secretion of various cytokines. In addition, it was confirmed that the signaling proteins were phosphorylated and cytokine production was induced by YC-1101 to enhance immunity.

### YC-1101 increased splenocyte proliferation and NK cell activity in CP-treated mice

**Fig. 3:**
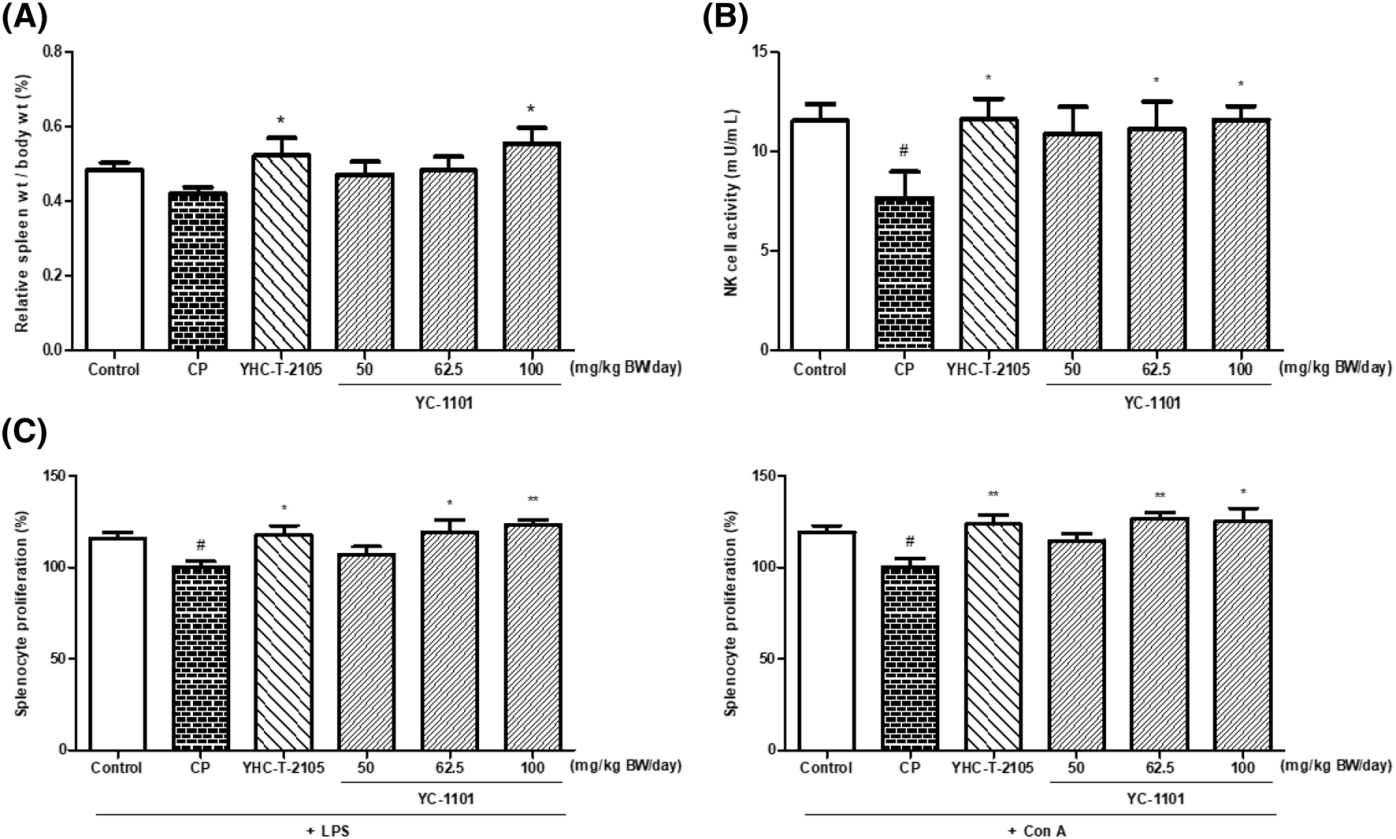
Effect of orally administered YC-1101 and YHC-T-2105 on spleen weight relative to body weight (**A**), NK cell activity (**B**), and splenocyte proliferation (**C**) in CP-treated mice. CP-treated mice were administered with YC-1101 (50, 62.5, and 100 mg/kg BW/day) and YHC-T-2105 (3 mg/g BW/day as a combination of ginsenoside Rg1, Rb1, and Rg3 in Korean red ginseng extract) for 14 days. Data are expressed as the mean ± SEM (n = 5–6). ^#^*p* < 0.05 vs. control group; *p < 0.05 vs. CP group; **p < 0.01 vs. CP group

CP-induced immunosuppression is a representative animal model used to assess immune enhancement in vivo. In this experiment, it was possible to confirm the immune-enhancing effects using biomarkers such as splenocyte proliferation and immune-related cytokine levels in the blood serum. Figure 3A shows spleen weight relative to body weight. YC-1101 treatment dose-dependently increased spleen weight relative to body weight compared to CP-treated mice. In particular, treatment with 100 mg/kg BW/day YC-1101 significantly increased the relative spleen weight compared to that of the CP-treated group, which was comparable to that of the YHC-T-2105-treated group (positive control), thereby indicating that YC-1101 can attenuate the myelosuppressive effect of CP on the spleen.

NK cells are white blood cells responsible for innate immunity. They mature in the liver and bone marrow (Liu et al., 2021). Because NK cells are immune cells that directly destroy virus-infected or cancer cells, they play an important role in the immune system (Björkström et al., 2022). NK cell activity was significantly lower in the CP-treated mice than in the control mice (Fig. 3B). Treatment of YC-1101 with 62.5 or 100 mg/kg BW/day and YHC-T-2105 significantly increased NK cell activity in CP-treated mice. In particular, the group treated with 100 mg/kg BW/day YC-1101 exhibited a similar effect on NK cell activity comparable to the YHC-T-2105-treated group.

As presented in Fig. 3C, the proliferation of splenocytes that responded to LPS or ConA stimulation was significantly inhibited in CP-treated mice compared with that in the control group. YC-1101-treated groups showed dose-dependent increases in splenocyte proliferation upon LPS and ConA stimulation. Moreover, oral administration of YC-1101 at the dosage of 62.5 and 100 mg/kg BW/day showed a significantly increased response of B cells comparable to that of the YHC-T-2105-treated group.

### YC-1101 activated immunomodulatory cytokine production in CP-treated mice

**Fig. 4:**
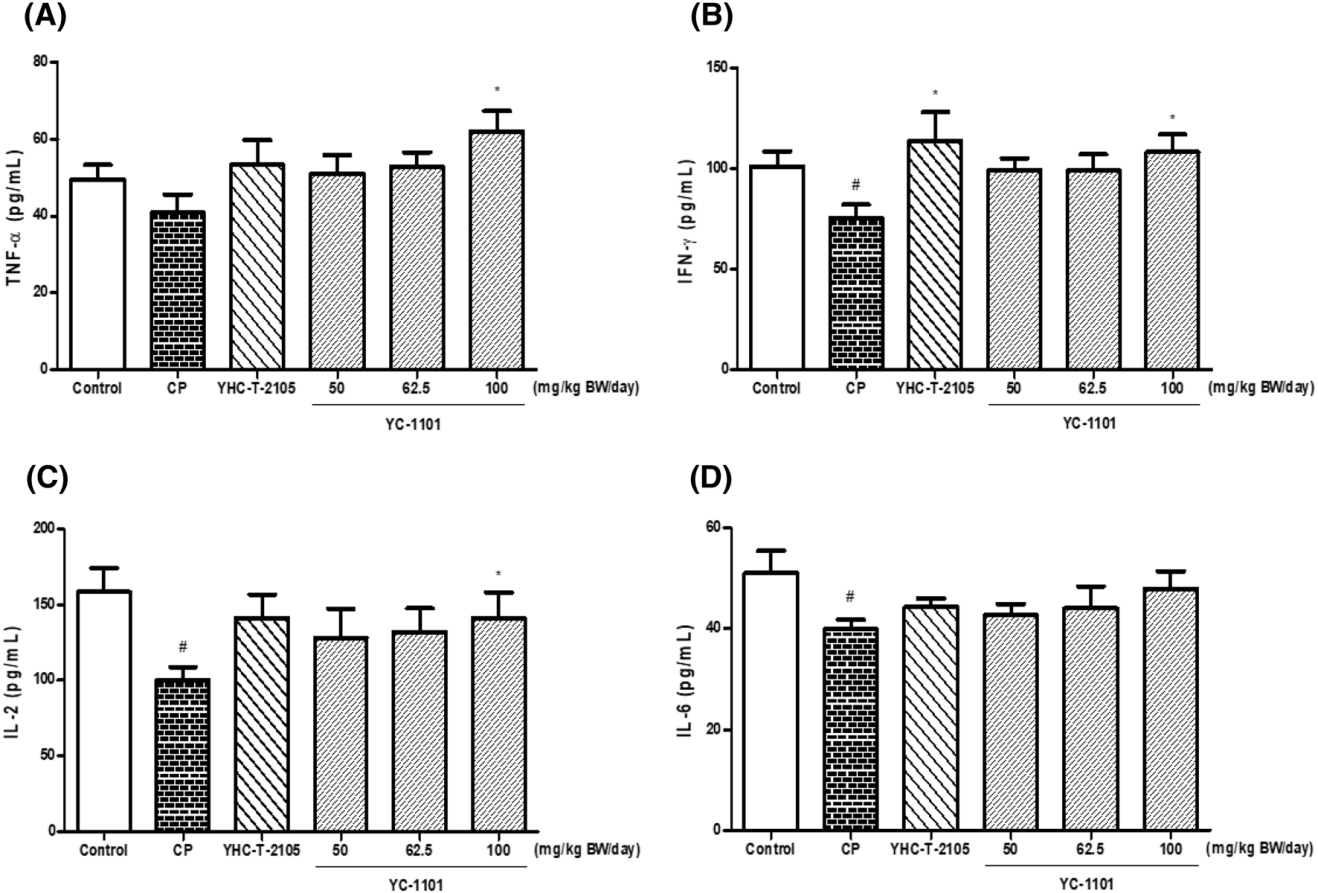
Effect of orally administered YC-1101 and YHC-T-2105 on the levels of TNF-α (**A**), IFN-γ (**B**), IL-2 (**C**), and IL-6 (**D**) in CP-treated mice. YC-1101 (50, 62.5, and 100 mg/kg BW/day) and YHC-T-2105 (3 mg/g BW/day as a combination of ginsenoside Rg1, Rb1, and Rg3 in Korean red ginseng extract) were administered orally once daily for 14 days. Mice were treated before samples administration orally 1–3 days and on day 13 via intraperitoneal injections of CP (70 mg/kg BW/day). The control group was treated with vehicle alone. Cytokines were assayed using commercial ELISA kits. Data are expressed as the mean ± SEM (n = 5–6). ^#^*p* < 0.05 vs. control group; *p < 0.05 vs. CP group

Figure 4 shows the effects of YC-1101 on cytokine production. In the CP treatment group, the cytokine levels were significantly reduced, indicating that CP successfully induced immunosuppression. As presented in Fig. 4B–D, the serum concentrations of IFN-γ, IL-2, and IL-6 were significantly lower in CP-treated mice than in control mice. YC-1101 treatment increased cytokine concentrations in CP-treated mice. In particular, treatment with 100 mg/kg BW/day YC-1101 significantly increased the concentrations of TNF-α, IFN-γ, and IL-2 compared with CP-treated mice, and the levels of IFN-γ, IL-2, and IL-6 were similar to those in the normal control group. Moreover, 100 mg/kg BW/day of YC-1101 increased the expression levels of TNF-α, IFN-γ, IL-2, and IL-6 by approximately 152%, 143%, 141%, and 120%, respectively, compared to those in CP-treated group. As a positive control, YHC-T-2105 also significantly increased the levels of IFN-γ compared to that in CP-treated mice.

In conclusion, based on the results of this study, we found that YC-1101 enhanced the secretion of various cytokines in macrophages and splenocytes by inducing the phosphorylation of AKT, ERK, and JNK. Oral administration of YC-1101 significantly alleviated cyclophosphamide-induced immunosuppression in mice. The highest dosage (100 mg/kg BW/day) of YC-1101 exhibited similar immunostimulatory effects by activating NK cell activity and various cytokines as the positive control. We believe that YC-1101 could be a potent and promising immune-enhancing natural product to support human health through immunosuppression.
